# Neonatal Cord Blood Oxylipins and Exposure to Particulate Matter in the Early-Life Environment: An ENVIR*ON*AGE Birth Cohort Study

**DOI:** 10.1289/EHP291

**Published:** 2016-11-04

**Authors:** Dries S. Martens, Sandra Gouveia, Narjes Madhloum, Bram G. Janssen, Michelle Plusquin, Charlotte Vanpoucke, Wouter Lefebvre, Bertil Forsberg, Malin Nording, Tim S. Nawrot

**Affiliations:** 1 Centre for Environmental Sciences, Hasselt University, Hasselt, Belgium; 2 Department of Chemistry, Umeå University, Umeå, Sweden; 3 MRC/PHE Centre for Environment and Health, School of Public Health, Imperial College London, London, England; 4 Belgian Interregional Environment Agency, Brussels, Belgium; 5 Flemish Institute for Technological Research (VITO), Mol, Belgium; 6 Division of Occupational and Environmental Medicine, Umeå University, Umeå, Sweden; 7 Department of Public Health and Primary Care, Leuven University, Leuven, Belgium

## Abstract

**Background:**

As part of the lipidome, oxylipins are bioactive lipid compounds originating from oxidation of different fatty acids. Oxylipins could provide a new target in the developmental origins model or the ability of early life exposure to change biology.

**Objectives:**

We studied the association between *in utero* PM_2.5_ (particulate matter with aerodynamic diameter < 2.5 μm) exposure and oxylipin profiles in newborns.

**Methods:**

Thirty-seven oxylipins reflecting the cyclooxygenase (COX), lipoxygenase (5-LOX and 12/15-LOX), and cytochrome P450 (CYP) pathways were assayed in 197 cord blood plasma samples from the ENVIR*ON*AGE birth cohort. Principal component (PC) analysis and multiple regression models were used to estimate associations of *in utero* PM_2.5_ exposure with oxylipin pathways and individual metabolites.

**Results:**

A principal component representing the 5-LOX pathway (6 metabolites) was significantly positively associated with PM_2.5_ exposure during the entire (multiple testing–adjusted *q*-value = 0.05) and second trimester of pregnancy (*q* = 0.05). A principal component representing the 12/15-LOX pathway (11 metabolites) was positively associated with PM_2.5_ exposure during the second trimester of pregnancy (*q* = 0.05). PM_2.5_ was not significantly associated with the COX pathway during any time period. There was a positive but nonsignificant association between second-trimester PM_2.5_ and the CYP pathway (*q* = 0.16).

**Conclusion:**

*In utero* exposure to particulate matter, particularly during the second trimester, was associated with differences in the cord blood levels of metabolites derived from the lipoxygenase pathways. These differences may indicate an effect of air pollution during *in utero* life on the inflammatory state of the newborn at birth. Oxylipins may be important mediators between early life exposures and health outcomes later in life.

**Citation:**

Martens DS, Gouveia S, Madhloum N, Janssen BG, Plusquin M, Vanpoucke C, Lefebvre W, Forsberg B, Nording M, Nawrot TS. 2017. Neonatal cord blood oxylipins and exposure to particulate matter in the early-life environment: an ENVIR*ON*AGE birth cohort study. Environ Health Perspect 125:691–698; http://dx.doi.org/10.1289/EHP291

## Introduction

Human exposure to particulate matter (PM) as a part of ambient air pollution has a substantial impact on human health outcomes such as cardiovascular morbidity and mortality and respiratory disease (Brunekreef and Holgate 2002; Nawrot et al. 2011). In 1990, Barker hypothesized that adult diseases are already programmed by the environment in fetal and infant life (Barker 1990). Indeed, recent findings suggest that indoor and outdoor exposure to environmental PM during *in utero* life is associated with adverse fetal outcomes including lower birth weight (Farmer et al. 2014; Glinianaia et al. 2004; Winckelmans et al. 2015). Underlying biological mechanisms involved in PM induced adverse health outcomes remain largely unknown, but indications of the involvement of inflammatory processes and oxidative stress are rising (Farmer et al. 2014; Proietti et al. 2013; Risom et al. 2005). PM-induced metabolic alterations might provide new insights into underlying biological pathways. Lipidomics elucidates complex structures and biological function of lipids (Shevchenko and Simons 2010), and might be important to understand the gene–environment–health paradigm. Unlike the genome, the lipidome (as part of the metabolome) is a very dynamic system that reflects the metabolic phenotype of the human body (Shevchenko and Simons 2010; Smilowitz et al. 2013). As part of the lipidome, oxylipins are bioactive lipid compounds originating from oxidation of different fatty acid (FA) precursors such as the ω-3 FAs (α-linolenic acid, docosahexaenoic acid, eicosapentaenoic acid) and ω-6 FAs (linoleic acid, arachidonic acid) (Zivkovic et al. 2012). Biologically active oxylipins are synthesized from these precursors primarily through the cyclooxygenase (COX), lipoxygenase (5-LOX and 12/15-LOX), and cytochrome P450 (CYP) enzymatic pathways. Oxylipin alterations have been studied in various disease contexts and may play important roles in acute and chronic inflammatory processes such as asthma, chronic bronchitis, chronic obstructive pulmonary disease, cystic fibrosis, and atherosclerosis (Conrad 1999; Eiserich et al. 2012; Lundström et al. 2011a; Tourdot et al. 2014). A growing body of knowledge in the field suggests that oxylipins may be used as biomarkers of metabolic health and disease (Zivkovic et al. 2011), and oxylipins may potentially be influenced by early life exposures. Both pro- and anti-inflammatory properties of oxylipins have been described, and environmental factors affecting these oxylipin levels remain a topic of interest (Caligiuri et al. 2014; Dennis and Norris 2015). There is limited information on the influence of air pollution on oxylipin pathways and metabolites, but recent experimental studies using mouse models of atherosclerosis suggest that exposure to ultrafine particles (UFP) (Li et al. 2013, 2015) and diesel exhaust (DE) (Yin et al. 2013) may increase levels of linoleic and arachidonic acid-derived oxylipins in different tissues and plasma. Oxylipin profiles in 18 nonasthmatic adults differed between bronchoalveolar lavage fluid (BALF) samples collected after exposure to subway air pollution versus ambient air (Lundström et al. 2011b). These findings indicate a potential role of oxylipins as relevant biomarkers in linking air pollution and adverse health outcomes.

The exposome encompasses the totality of environmental and external exposures, including exposure to combustion particles in the environment and subsequent physiological interaction with the genome, proteome and metabolome (Rappaport and Smith 2010; Wild 2012). The “pregnancy exposome” is important both as the starting point of the life-course exposome and because of the high vulnerability of the fetus to effects of environmental factors. Here, we studied associations of cord blood plasma oxylipin profiles with *in utero* exposure to air pollution (PM_2.5_; exposure with aerodynamic diameter < 2.5 μm) as a potentially important component of the pregnancy exposome. We measured 37 different oxylipins from the three main biosynthetic pathways (COX, LOX, and CYP) to study the potential influence of *in utero* PM_2.5_ exposure on these pathways and individual metabolites in the newborn.

## Methods

### Study Population and Data Collection

This study is part of the ongoing ENVIR*ON*AGE (ENVIRonmental influence *ON* AGEing in early life) birth cohort and complies with the Declaration of Helsinki and was approved by the Ethics Committee of Hasselt University and East-Limburg Hospital in Genk, Belgium. For the present study, we included 222 mother–newborn pairs (only singletons) from the ENVIR*ON*AGE birth cohort, recruited between Friday 1200 hours and Monday 0700 hours from February 2010 through April 2014 from the East-Limburg hospital in Genk. Twenty individuals were excluded because cord blood plasma samples showed hemolysis, 4 were excluded because of a lack of exposure data, and 1 was excluded because of missing information on maternal smoking, resulting in a total study population of 197 individuals. The catchment area of the hospital included the province Limburg in Flanders, Belgium, and combines both urban and suburban to rural areas with population densities between the municipalities ranging from 82 to 743 inhabitants/km^2^. The participation rate of eligible mothers in the birth cohort (mothers able to fill out a Dutch language questionnaire) was 61% and enrollment was spread over all seasons of the year. Participating mothers provided written informed consent when they arrived at the hospital for delivery. They completed study questionnaires in the postdelivery ward to provide detailed information on maternal age, pregestational body mass index (BMI), maternal education, occupation, smoking status, alcohol consumption, place of residence, use of medication, parity, and the newborn’s ethnicity. Former smokers were defined as those who had quit smoking before pregnancy. Smokers were those who continued smoking during pregnancy. Based on the native country of the newborn’s grandparents, we classified his/her ethnicity as European-Caucasian when two or more grandparents were European or non-European when at least three grandparents were of non-European origin. Maternal education was coded as “low” (no diploma or primary school), “middle” (high school), or “high” (college or university degree). The ENVIR*ON*AGE birth cohort did not differ from all births in Flanders, Belgium, as to maternal age, education, gender, ethnicity, and birth weight (see Table S1) (Cox et al. 2013); but compared with all births in Flanders, our population was more likely to be primiparous (56% vs. 47%) and less likely to have had three or more previous births (10% vs. 18%). Cord blood samples were collected immediately after delivery, along with perinatal parameters such as birth date, gestational age, the newborn’s gender, birth weight, and length, Apgar score, pH of arterial cord blood, and ultrasonographic data. Immediately after birth, all neonates were assessed for congenital anomalies and were considered healthy when the Apgar score after 5 min ranged between 7 and 10.

### Exposure Assessment

Regional background levels of PM_2.5_ (μg/m^3^) were interpolated for each mother’s residential address separately using a spatial temporal interpolation method (kriging) (Janssen et al. 2008). This method uses pollution data collected in the official fixed-site monitoring networks (*n* = 34) and land cover data obtained from satellite images (CORINE land cover data set; http://www.eea.europa.eu/publications/COR0-landcover) in combination with a dispersion model as described by Lefebvre et al. (2011, 2013). This model chain provides daily PM_2.5_ values using data both from the Belgian telemetric air quality network, point sources, and line sources which then are interpolated to a high-resolution receptor grid (25 × 25 m). The interpolation tool explained > 80% of the temporal and spatial variability (*R* ^2^) in the Flemish region of Belgium (Maiheu et al. 2013). Total PM_2.5_ exposure for entire pregnancy duration was calculated as the mean PM_2.5_ concentration (μg/m^3^) of all pregnancy days. Date of conception was estimated based on ultrasonographic data (Janssen et al. 2012). Additionally, we calculated mean PM_2.5_ (μg/m^3^) concentrations during each trimester of pregnancy (1–13 weeks, 14–26 weeks, and 27 weeks until delivery). Address changes for 24 mothers (12%) who moved residence during pregnancy were taken into account when calculating exposures during the different time windows.

### Sample Collection and Oxylipin Profiling by UPLC-ESI-MS/MS

Umbilical cord blood (mixture of arterial and venous blood) was collected directly after delivery in BD Vacutainer® plastic whole blood tubes with spray-coated K2EDTA (BD, Franklin Lakes, NJ, USA). Within 20 min after cord blood collection, samples were centrifuged at 3,200 rpm for 15 min to separate plasma from blood. Plasma was collected and stored in Eppendorf® tubes at –80°C until analysis. Cord blood plasma levels of total cholesterol and high-density lipoprotein (HDL) was measured using a Roche cobas c701 system (F. Hoffmann-La Roche AG, Basel, Switzerland). Oxylipin analysis was performed as previously described and validated (Gouveia-Figueira et al. 2015). Native, deuterated internal standards (IS) and the recovery standard CUDA (12-{[(cyclohexylamino) carbonyl]amino}-dodecanoic acid) were purchased from Cayman Chemical (Ann Arbor, MI, USA) or Larodan Fine Chemicals AB (Malmö, Sweden) (see “Chemical details for UPLC-MS/MS” in the Supplemental Material for details). All solvents were of HPLC grade or higher. A Milli-Q Gradient system (Millipore, Milford, MA, USA) was used to purify water used in UPLC mobile phase. In brief, oxylipins were extracted using a solid phase extraction (SPE) procedure on 60 mg Waters Oasis-HLB cartridges (Milford, MA, USA). Cartridges were washed and then preconditioned with MilliQ water buffer (5% methanol, 0.1% acetic acid). Next, 750 μL of cord plasma together with 10 μL antioxidant solution [0.2 mg/mL butylhydroxytoluene, EDTA in methanol/water (1:1)] and 10 μL of internal standard mixture (400 nM/standard, 12,13-DiHOME-d_4_, 9-HODE-d_4_, PGE_2_-d_4_, TXB_2_-d_4_) solution were applied on the cartridge. Cartridges were rinsed with 2 × 2 mL buffer and oxylipins were then eluted using 2 mL methanol and 2 mL ethyl acetate in tubes containing 6 μL 30% glycerol solution in methanol. Next, solvents were evaporated using a SpeedVAC system (Farmingdale, NY, USA). Residues were reconstituted in 100 μL methanol and the solution was transferred into LC-vials containing 10 μL recovery standard solution (5 ng/mL CUDA).

Profiling of oxylipins was performed by injecting 10 μL of the solution in the LC-vial on an Agilent 6490 triple quadrupole UPLC-MS/MS system equipped with the iFunnel Technology (Agilent Technologies, Santa Clara, CA, USA) operating in negative mode. An Acquity UPLC BEH C_18_ column (Waters) with in-line filter (2.1 × 150 mm, 130 Å, 1.7 μm particle size) was used at 40°C for chromatographic separation using a constant flow rate of 0.3 mL/min. The mobile phase consisted of *a*) 0.1% acetic acid in MilliQ water and *b*) acetonitrile:isopopanol (90:10) with the following gradient for acetonitrile:isopopanol (90:10): 0.0–3.5 min 10–35%, 3.5–5.5 min 40%, 5.5–7.0 min 42%, 7.0–9.0 min 50%, 9.0–15.0 min 65%, 15.0–17.0 min 75%, 17.0–18.5 min 85%, 18.5–19.5 min 95%, 19.5–21 min 10%, 21.0–25.0 min 10%. Peak identification and manual integration was performed using the MassHunter Workstation Software (Agilent Technologies). The stable isotope dilution method was used to quantify each metabolite using calibration curves of ratios of peak areas of native compounds by IS peak area (see Tables S2 and S3 for IS assignment to each native standard and calibration curves concentrations respectively). The recovery rates (%, mean ± SD, *n*) of the internal standards in the present study were as follows: TXB_2_-d_4_ (68 ± 18, *n* = 97), 12(13)-DiHOME-d_4_ (90 ± 13, *n* = 197), 9(S)-HODE-d_4_ (76 ± 19, *n* = 197), and PGE_2_-d_4_ (85 ± 23, *n* = 197). Samples with analyte peak areas below the limit of detection (LOD) were assigned a value of LOD/2 [LOD values were previously reported and are provided in Table S4 (Gouveia-Figueira et al. 2015)].

### Statistical Analysis

**Figure 1:**
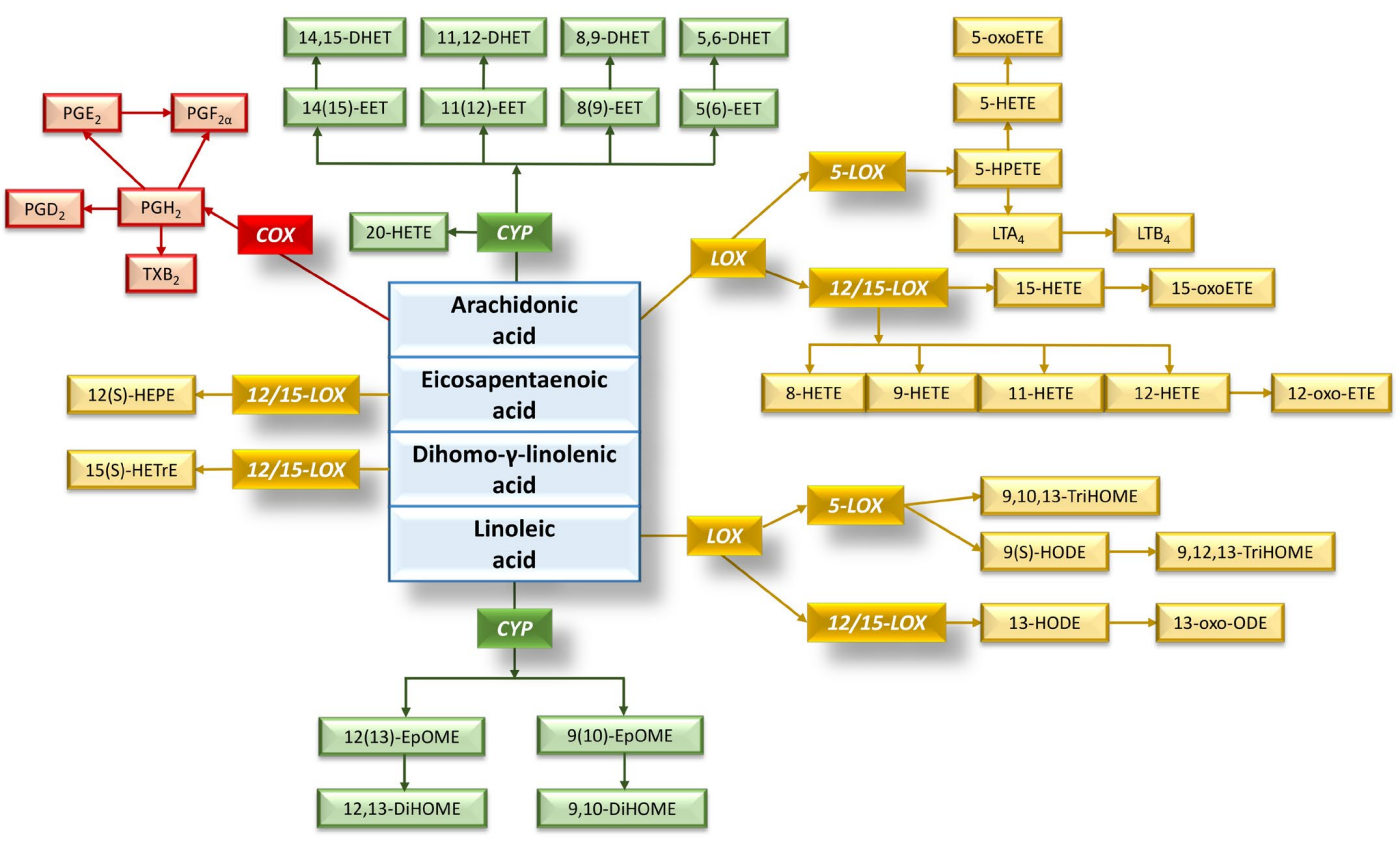
Metabolic scheme of targeted oxylipins. Oxylipin synthesis via different biosynthetic pathways, COX, 5-LOX, 12/15-LOX, and CYP from their corresponding precursor fatty acids, arachidonic acid, eicosapentaenoic acid, dihomo-γ-linolenic acid, and linoleic acid (Zivkovic et al. 2012). All shown metabolites were targeted except for 5-HPETE (5-hydroperoxy-eicosatetraenoic acid), LTA_4_ (Leukotriene A4), and PGH_2_ (prostaglandin H2) and classified by biosynthetic pathway. Nondetectable targeted oxylipins (17-HDoDE, Resolvin D1, and Resolvin D2) are not shown on the scheme. A list of full metabolite names is provided in Table S4.

For database management and statistical analysis, we used SAS 9.3 software (SAS Institute Inc., Cary, NC, USA). Metabolite concentrations were log10 transformed to approximate the normal distribution. For logarithmically transformed variables, we report central tendency and spread of data as geometric mean and interquartile range, respectively. Based on different pathways involved in oxylipin biosynthesis, metabolites were classified by the main biosynthetic pathways COX, CYP, 5-LOX, and 12/15-LOX (Figure 1; see also Table S4) (Zivkovic et al. 2012). We combined metabolites originating from the same pathway into different principal components using the PROC PINCOMP procedure in SAS 9.3. We derived unadjusted Pearson correlations for the first principal component of each pathway and PM_2.5_ during each time window. In our main analyses, we used separate multi-variable adjusted principal component regressions to estimate associations between *in utero* PM_2.5_ exposures and the oxylipin pathways, using the first principal components as the dependent variables in these models. The multi-variable–adjusted models were adjusted for gestational age, pregestational BMI, and maternal age as continuous variables; maternal smoking status (never, ex-smoker, current), maternal education (low, middle, high); newborn gender; cord blood total cholesterol and HDL concentrations (continuous); batch; and sample storage time. Due to correlations between different exposure windows (trimesters of pregnancy) of PM_2.5_, models of trimester-specific associations were additionally adjusted for PM_2.5_ exposure during the other trimesters. We applied the Benjamini–Hochberg false discovery rate (FDR) correction for multiple testing (represented by a *q*-value) based on the four different pathways and four different exposure windows tested (total number of tests = 16). Pathway models were classified as significant when *q*-values where ≤ 0.05. Individual metabolites from significantly associated pathways revealed in the main analysis by principal component regression were tested in a secondary analysis for PM_2.5_ dependency during the different significant exposure windows using multiple linear regression models. Estimates (beta-coefficients) of the principal component regression models and percent change in individual metabolite concentration (nM) are presented for each 5 μg/m^3^ increase of PM_2.5_. Q-Q plots of the residuals showed a normal distribution (data not shown).

### Sensitivity Analyses

We performed the following sensitivity analyses for pathways with significant associations with PM_2.5_ during at least one exposure time window: models additionally adjusted for third-trimester apparent temperature (based on tertiles of third-trimester apparent temperature to account for potential non-linearity); models additionally adjusted for trimester-specific average NO_2_ concentrations; models that included PM_2.5_ concentrations during only one trimester; models that excluded women with a cesarean (C-) section or one of the following pregnancy complications: diabetes, hypertension, hypo- and hyperthyroidism, and mothers with an infection during pregnancy (*n* = 18); models that were not adjusted for gestational age; and models stratified by newborn gender. In addition, for each significant pathway, we modeled principal components that were derived after excluding unstable metabolites (defined as metabolites that were significantly associated with storage time) and metabolites with > 40% of sample concentrations < LOD.

## Results

### Characteristics of the Study Population and Exposure Levels

General characteristics of the study population consisting of 197 mother–newborn pairs are presented in Table 1. Data presented are complete for all variables other than cord blood pH (*n* = 177). Average maternal age was 29.1 years and ranged from 19 to 44 years. Mothers had an average (± SD) pregestational BMI of 23.8 ± 4.7 kg/m^2^. Most women (70%, *n* = 137) never smoke cigarettes, and 44 women (22%) stopped smoking before pregnancy, whereas 16 mothers (8%) continued smoking during pregnancy. About 85% (*n* = 168) of the newborns were Europeans of Caucasian ethnicity. Five minutes after delivery, > 90% of the newborns had an Apgar score of 9 or 10. The newborns, among them 89 girls (45.2%) and 108 boys (54.8%), had a mean gestational age of 39.3 weeks (range, 33–41 weeks) and comprised 111 (56%) primiparous and 66 (34%) secundiparous newborns. Birth weight averaged 3,453 ± 420 g, ranging from 2,135 to 4,445 g. Among the newborns, 10 (5.1%) were preterm births (gestational age < 37 weeks) and 3 (1.5%) had a low birth weight (< 2,500 g). Mean cord blood plasma total cholesterol and HDL levels were, respectively, 69 ± 18 mg/dL and 31 ± 11 mg/dL. Among the mothers, 11 (5.6%) were diagnosed with gestational diabetes, 3 (1.5%) had hypertension, 1 mother (0.5%) had hypothyroidism, and 3 (1.5%) had an infection during pregnancy. In total 7 mothers (3.6%) had a C-section. Of the 18 mothers who experienced a pregnancy complications, 2 had a C-section. Cord blood plasma oxylipins were analyzed in three batches within 3 consecutive days and the average storage time for cord blood plasma samples was 84.6 ranging from 7.3 to 218.7 weeks. Table 2 displays the daily outdoor PM_2.5_ exposure levels averaged for each of the three trimesters of pregnancy. Average PM_2.5_ concentrations for the first, second, and third trimester of pregnancy were 15.5 ± 5.4 μg/m^3^, 15.4 ± 5.1 μg/m^3^, and 16.1 ± 5.2 μg/m^3^ respectively. During entire pregnancy, PM_2.5_ exposure concentrations averaged 15.7 ± 2.6 μg/m^3^. First- and third-trimester PM_2.5_ concentrations were inversely correlated (*r* = –0.57, *p* < 0.0001), whereas first- and third-trimester PM_2.5_ concentrations did not correlate with second-trimester PM_2.5_ concentrations (*r* = 0.09, *p* = 0.22 and *r* = 0.08, *p* = 0.26, respectively).

**Table 1:** Characteristics of mother–newborn pairs (*n* = 197) selected from the ENVIR*ON*AGE birth cohort between February 2010 and April 2014.

Characteristic	Mean ± SD or *n* (%)
Maternal
Age (years)	29.1 ± 4.8
Education
Low	27 (13.7)
Middle	76 (38.6)
High	94 (47.7)
Smoking status
Never-smoker	137 (69.5)
Stopped smoking before pregnancy	44 (22.4)
Continued smoking during pregnancy	16 (8.1)
Alcohol consumption during pregnancy
No	167 (84.8)
Pregestational BMI (kg/m^2^)	23.8 ± 4.7
Parity
1	111 (56.4)
2	66 (33.5)
≥ 3	20 (10.1)
Cesarean section	7 (3.6)
Pregnancy complications
Gestational diabetes	11 (5.6)
Hypertension	3 (1.5)
Hypo-/hyperthyroidism	1 (0.5)
Infection	3 (1.5)
Newborn
Gender
Boys	108 (54.8)
Ethnicity
European-Caucasian	168 (85.3)
Gestational age (weeks)	39.3 ± 1.4
Apgar score 5 min after birth
7	3 (1.5)
8	15 (7.6)
9	61 (31.0)
10	118 (59.9)
Season of delivery
Winter	59 (30)
Spring	37 (19)
Summer	52 (26)
Autumn	49 (25)
pH artery cord blood	7.3 ± 0.07
Birth weight (g)	3453.6 ± 420.7
Total cholesterol (mg/dL)	69.0 ± 18.0
HDL level (mg/dL)	30.6 ± 10.8
Complete data for all variables except for pH artery cord blood (*n* = 177).	

**Table 2:** Outdoor PM_2.5_ (μg/m^3^) exposure characteristics for the 197 mothers included in this study from the ENVIR*ON*AGE birth cohort.

Exposure window	Mean ± SD	25th percentile	75th percentile
Trimester 1	15.5 ± 5.4	11.4	19.9
Trimester 2	15.4 ± 5.1	11.1	19.7
Trimester 3	16.1 ± 5.2	12.0	19.8
Entire pregnancy	15.7 ± 2.6	13.5	17.5

### Oxylipin Levels in Newborns

We targeted 37 different individual oxylipins in cord blood plasma and detected 34 of them. Resolvin D1, Resolvin D2, and 17-HDoHE were not detected above the LOD in any of the samples, so we excluded these metabolites from further statistical analysis. Mean cord blood plasma oxylipin concentrations (nM) are shown in Table S4. For 21 metabolites all samples were detected above the LOD, and for another 10 metabolites ≥ 94% of the samples were detected above the LOD (see Table S4). LTB_4_ was detected in only 28% of the samples, 12-oxoETE was detected in only 56% of the samples, and 9-HETE was detected in 77% of the samples. Three metabolites (9,10,13-TriHOME, 9,12,13-TriHOME, and TXB_2_) showed a significant negative association with storage time and were classified as unstable (data not shown).

### Cord Blood Oxylipin Pathways in Association with Maternal PM_2.5_ Exposure

**Figure 2:**
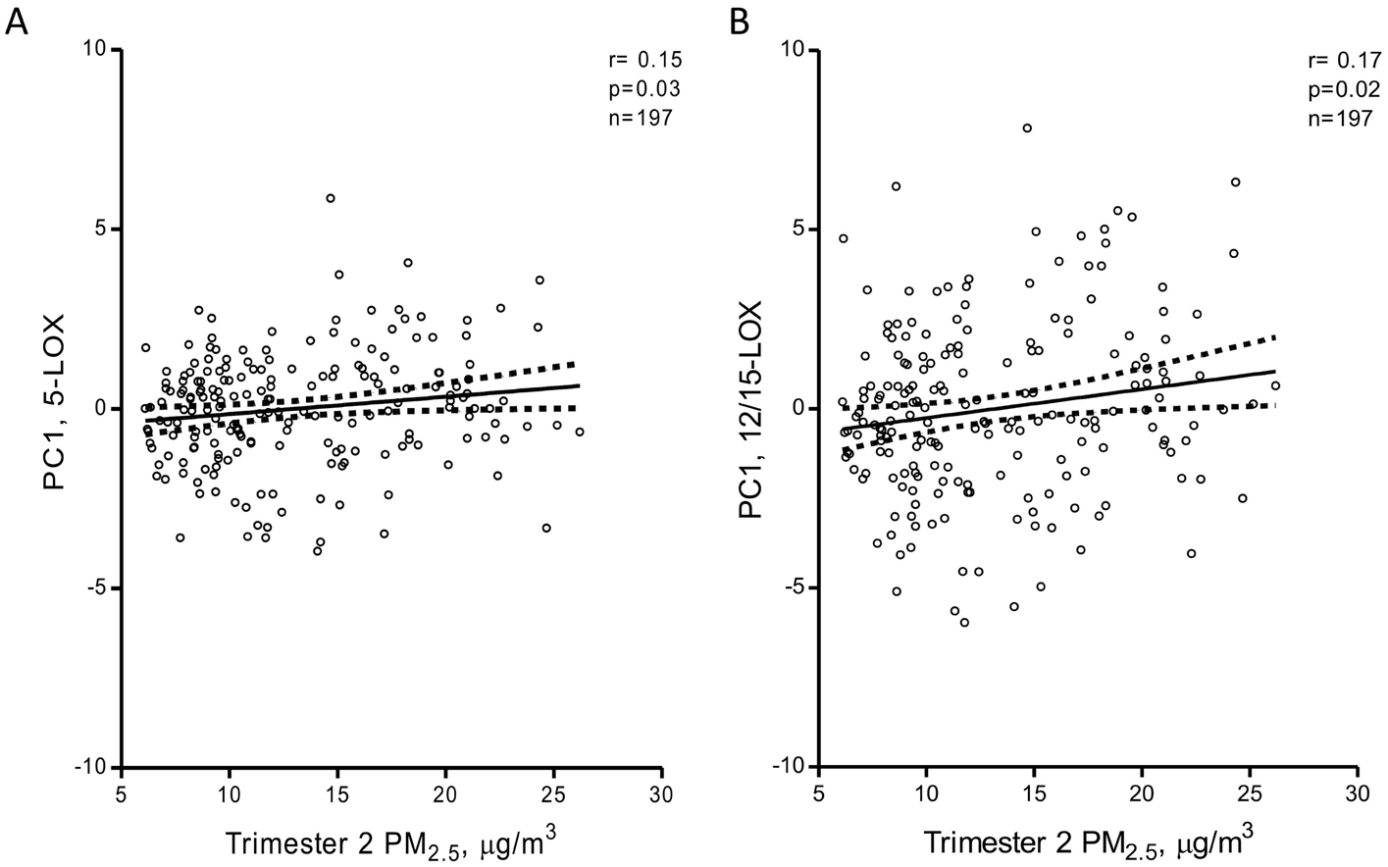
Unadjusted Pearson correlation plots with 95% confidence intervals between oxylipin pathways and PM_2.5_ exposure during the second trimester: (*A*) correlation with the 5-LOX pathway and (*B*) correlation with the 12/15-LOX pathway. PC1, first principal component.

**Figure 3:**
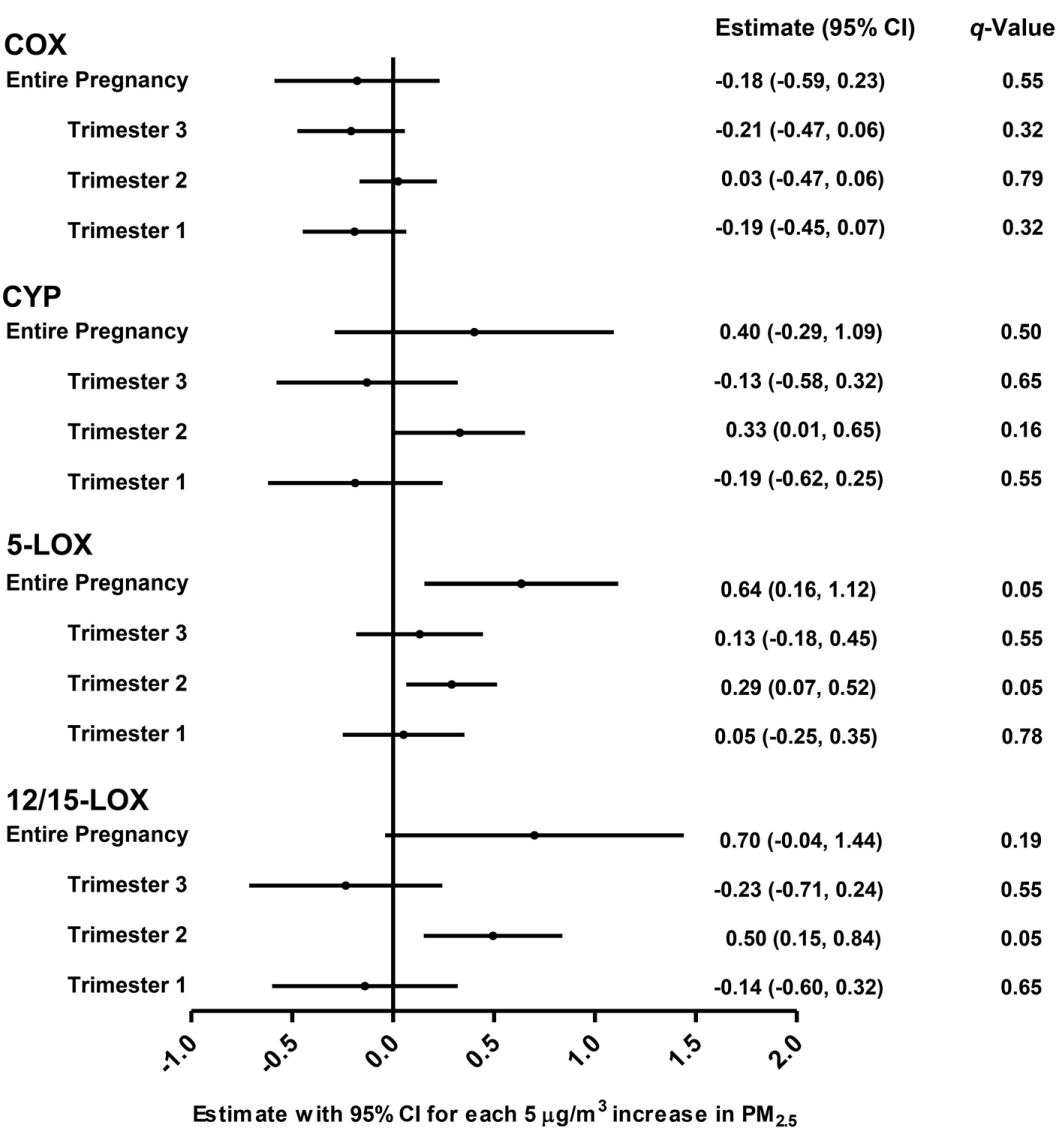
Association between oxylipin biosynthetic pathways and *in utero* PM_2.5_ exposure during different time windows. Estimates are presented for each 5-μg/m^3^ increase in PM_2.5_. Models were adjusted for gestational age, pregestational BMI, maternal age, maternal smoking status, maternal education, newborn gender, cord blood total cholesterol and HDL levels, batch, and sample storage time. Trimester-specific windows were additionally adjusted for the other PM_2.5_ exposure windows (trimesters). Q indicates the multiple testing adjusted *p*-values using the Benjamini–Hochberg procedure.

A heat map illustrating the correlations between the individual metabolites in each of the four studied oxylipin pathways is provided in Figure S1. For each oxylipin biosynthetic pathway (COX, CYP, 5-LOX, 12/15-LOX; see Figure 1 and also Table S4 for assignment), principal components were derived as linear combinations of different metabolites involved in each pathway. The first principal components explained 42–55% of the variance for the four individual pathways, and factor loadings were ≥ 0.54 for 28 of the 34 individual metabolites (see Table S5). Characteristics of the first principal components used in the multi-variable–adjusted principal component regression models are shown in Table S5. Both before (Figure 2) and after adjustment for gestational age, pregestational BMI, maternal age, maternal smoking status, maternal education, newborn gender, cord blood total cholesterol and HDL levels, batch and storage time in multi-variable–adjusted models, the 5-LOX and 12/15-LOX oxylipin pathways were positively and significantly (*q* ≤ 0.05) associated with *in utero* exposure to PM_2.5_ during the second trimester of pregnancy (Figure 3). The 5-LOX pathway also was positively associated with PM_2.5_ exposure during the entire pregnancy (*q* = 0.05), whereas the association with the 12/15-LOX pathway was positive but not significant (*q* = 0.19). Positive but nonsignificant associations were also estimated for the principal component reflecting the CYP pathway and PM_2.5_ exposure during the second trimester (*q* = 0.16) and the entire pregnancy (*q* = 0.5) (Figure 3). The principal component reflecting the COX pathway was not significantly associated with PM_2.5_ exposure during any time period. Results of the sensitivity analyses for 5-LOX and exposure during the second trimester and entire pregnancy, and for 12/15-LOX and exposure during the second trimester, were generally consistent with the main analyses (Table 3).

**Table 3:** Sensitivity analyses for the 5-LOX and 12/15-LOX pathways in association with *in utero* PM_2.5_ exposure.

Analysis	Trimester 1 PM_2.5_		Trimester 2 PM_2.5_		Trimester 3 PM_2.5_		Entire pregnancy PM_2.5_	
	β (95% CI)	*p*‑Value	β (95% CI)	*p*‑Value	β (95% CI)	*p*‑Value	β (95% CI)	*p*‑Value
5-LOX
Main model	0.05 (–0.25, 0.35)	0.73	0.29 (0.07, 0.52)	0.01	0.13 (–0.18, 0.45)	0.41	0.64 (0.16, 1.12)	0.01
Apparent temperature adjusted	0.02 (–0.40, 0.44)	0.93	0.28 (0.01, 0.55)	0.04	0.12 (–0.26, 0.50)	0.53	0.67 (0.14, 1.19)	0.01
NO_2_ adjusted	–0.12 (–0.62, 0.37)	0.62	0.32 (–0.02, 0.67)	0.06	0.30 (–0.12, 0.72)	0.16	0.70 (0.06, 1.33)	0.03
Single-trimester PM_2.5_	–0.01 (–0.23, 0.21)	0.94	0.30 (0.08, 0.52)	0.008	0.11 (–0.12, 0.34)	0.35	NA	NA
Boys (*n* = 108)	–0.06 (–0.47, 0.35)	0.78	0.34 (0.03, 0.65)	0.03	0.12 (–0.29, 0.53)	0.57	0.65 (0.00, 1.30)	0.05
Girls (*n* = 89)	0.38 (–0.18, 0.93)	0.18	0.31 (–0.06, 0.68)	0.10	0.31 (–0.25, 0.88)	0.28	0.98 (0.21, 1.75)	0.01
Not adjusted for gestational age	0.06 (–0.24, 0.35)	0.71	0.29 (0.07, 0.51)	0.01	0.14 (–0.17, 0.44)	0.38	0.63 (0.16, 1.11)	0.009
Excluding pregnancy complications and C‑sections (*n* = 174)	0.09 (–0.23, 0.41)	0.58	0.27 (0.04, 0.50)	0.02	0.18 (–0.14, 0.51)	0.27	0.66 (0.17, 1.15)	0.009
Excluding TriHOMEs and LTB_4_^*a*^	0.04 (–0.24, 0.31)	0.80	0.26 (0.06, 0.47)	0.01	0.03 (–0.26, 0.31)	0.86	0.52 (0.08, 0.95)	0.02
12/15-LOX
Main model	–0.14 (–0.60, 0.32)	0.55	0.50 (0.15, 0.84)	0.005	–0.23 (–0.71, 0.24)	0.34	0.70 (–0.04, 1.44)	0.06
Apparent temperature adjusted	–0.34 (–0.98, 0.30)	0.30	0.38 (–0.03, 0.78)	0.07	–0.03 (–0.60, 0.55)	0.92	0.64 (–0.16, 1.44)	0.12
NO_2_ adjusted	–0.44 (–1.19, 0.31)	0.25	0.56 (0.04, 1.08)	0.03	–0.19 (–0.83, 0.46)	0.57	0.83 (–0.15, 1.82)	0.10
Single-trimester PM_2.5_	0.05 (–0.29, 0.39)	0.77	0.47 (0.13, 0.81)	0.007	–0.11 (–0.46, 0.25)	0.56	NA	NA
Boys (*n* = 108)	–0.24 (–0.91, 0.42)	0.47	0.56 (0.06, 1.06)	0.03	–0.08 (–0.76, 0.59)	0.81	0.78 (–0.27, 1.84)	0.14
Girls (*n* = 89)	–0.03 (–0.79, 0.73)	0.93	0.52 (0.01, 1.03)	0.04	–0.40 (–1.17, 0.37)	0.31	0.91 (–0.18, 1.99)	0.10
Not adjusted for gestational age	–0.08 (–0.54, 0.37)	0.72	0.48 (0.13, 0.82)	0.007	–0.17 (–0.65, 0.30)	0.47	0.73 (0.00, 1.47)	0.05
Excluding pregnancy complications and C‑sections (*n* = 174)	–0.08 (–0.59, 0.42)	0.74	0.46 (0.10, 0.82)	0.01	–0.12 (–0.62, 0.39)	0.65	0.74 (–0.02, 1.51)	0.06
Excluding 12-oxoETE^*b*^	–0.14 (–0.59, 0.32)	0.56	0.48 (0.14, 0.82)	0.007	–0.23 (–0.71, 0.24)	0.34	0.67 (–0.07, 1.40)	0.08
Beta-coefficients are presented for each 5-μg/m^3^ increase in PM_2.5_. Unless otherwise indicated, all models were adjusted for gestational age, pregestational BMI, maternal age, maternal smoking status, maternal education, newborn gender, cord blood total cholesterol and HDL levels, batch, and sample storage time. ^***a***^Exclusion of unstable 9,10,13-TriHOME and 9,12,13-TriHOME due to storage time dependency and LTB_4_ due to 72% of the samples under the limit of detection (LOD). ^***b***^Exclusion of 12-oxoETE because 44% of the samples were below the LOD.								

### Individual Cord Blood Oxylipins Associated with Maternal PM_2.5_ Exposure

Associations between all metabolites (independent of pathways) with exposure during all different pregnancy time windows are summarized in Figure S2 and Table S6. After identification of aforementioned significantly associated oxylipin pathways with *in utero* exposure to PM_2.5_ during the second trimester and entire pregnancy, we further examined individual metabolites of the 5-LOX and 12/15-LOX pathways in association with these significant exposure windows (Table 4). Positive associations of 3 profiled 5-LOX metabolites [5-HETE, *p* = 0.03; 5-oxoETE, *p* = 0.03; 9(S)-HODE, *p* = 0.06] were estimated in relation with *in utero* PM_2.5_ exposure during second trimester. Exposure during the entire pregnancy was positively associated with the following 5-LOX metabolites: 5-HETE, *p* = 0.05; 5-oxoETE, *p* = 0.006; 9,10,13-TriHOME, *p* = 0.08; and 9,12,13-TriHOME, *p* = 0.05. Eight of the 11 targeted individual linoleic (13-HODE, *p* = 0.05), arachidonic acid (8-HETE, *p* = 0.02; 11-HETE, *p* = 0.02; 12-HETE, *p* = 0.02; 12-oxoETE, *p* = 0.03 and 15-HETE, *p* = 0.006), eicosapentaenoic acid [12(S)-HEPE, *p* = 0.02], and dihomo-γ-linolenic [15(S)-HETrE, *p* = 0.04] derived metabolites from the 12/15-LOX pathway were positively associated with *in utero* exposure to PM_2.5_ during the second trimester of pregnancy.

**Table 4:** Estimated percent difference in individual metabolite concentration (nM) from the 5-LOX and 12/15-LOX pathways in association with *in utero* PM_2.5_ exposure.

Pathway/metabolite	Loading on first PC	Trimester 2 PM_2.5_		Entire pregnancy PM_2.5_	
		% difference (95% CI)^*a*^^,^^*b*^^,^^*c*^	*p*-Value	% difference (95% CI)^*a*^^,^^*b*^	*p*-Value
5-LOX
5-HETE	0.62	11.7 (1.4, 23.1)	0.03	22.5 (–0.4, 50.7)	0.05
5-oxoETE	0.61	20.3 (1.7, 42.4)	0.03	66.0 (16.2, 137.3)	0.006
9(S)-HODE	0.76	11.9 (–0.3, 25.6)	0.06	17.2 (–8.4, 50.0)	0.20
LTB_4_	0.05	–1.8 (–13.2, 11.0)	0.77	3.0 (–20.9, 34.2)	0.82
9,10,13-TriHOME	0.76	8.5 (–3.2, 21.5)	0.16	24.6 (–2.2, 58.9)	0.08
9,12,13-TriHOME	0.81	9.5 (–1.7, 21.9)	0.10	25.6 (–0.2, 58.0)	0.05
12/15-LOX
8-HETE	0.83	15.3 (2.4, 29.8)	0.02	NA	NA
9-HETE	0.31	16.1 (–8.8, 47.7)	0.22	NA	NA
11-HETE	0.93	14.0 (2.3, 27.1)	0.02	NA	NA
12-HETE	0.83	28.7 (4.7, 58.2)	0.02	NA	NA
12(S)-HEPE	0.63	27.9 (4.7, 56.2)	0.02	NA	NA
12-oxoETE	0.55	49.7 (3.2, 117.1)	0.03	NA	NA
15-HETE	0.90	16.3 (4.5, 29.4)	0.006	NA	NA
15(S)-HETrE	0.88	15.7 (0.8, 32.7)	0.04	NA	NA
15-oxoETE	0.64	10.3 (–3.9, 26.7)	0.16	NA	NA
13-HODE	0.81	11.4 (–0.2, 24.3)	0.05	NA	NA
13-oxoODE	0.59	8.0 (–5.8, 23.8)	0.27	NA	NA
first PC, first principal component. ^***a***^Effect size was estimated for an increase of 5 μg/m^3^ of PM_2.5_. ^***b***^Models adjusted for gestational age, pregestational BMI, maternal age, maternal smoking status, maternal education, newborn gender, cord blood total cholesterol and HDL levels, batch and sample storage time. ^***c***^Additional adjustment for the other PM_2.5_ exposure windows (trimesters). NA as the 12/15-LOX pathway is not significant for this exposure window.					

## Discussion

The key finding of our paper is that *in utero* exposure to PM_2.5_ during pregnancy is associated with levels of free circulating oxylipins in cord blood plasma, particularly oxylipins from the lipoxygenase pathway (5-LOX and 12/15-LOX). Several individual cord blood oxylipins included in the 5-LOX and 12/15-LOX pathways were associated with *in utero* exposure to PM_2.5_ during the second trimester. To our knowledge, this is the first extensive report on targeting 37 oxylipins in cord blood simultaneously by LC/MS-MS quantification. These findings elucidate new early-life effects of air pollution on the metabolomic level, more specifically at the level of oxylipins.

To date, evidence concerning PM-induced alterations of circulating oxylipin metabolites is limited to experimental findings in mice. Apolipoprotein E–deficient mice exposed to diesel exhaust for 2 weeks showed increased concentrations of oxylipins (5-HETE, 12-HETE, 15-HETE, and 13-HODE) targeted from the 5-LOX and 12/15-LOX pathway compared with FA-exposed mice. Plasma levels of 12-HETE and 13-HODE were significantly elevated in exposed mice compared with the FA controls, and in liver and large intestine tissue only 5-HETE showed increased concentrations in the exposed mice. In BALF samples, both higher levels of 12-HETE and 15-HETE were measured in the DE-exposed mice compared with the FA controls, showing different elevated oxylipins in different tissues (Yin et al. 2013). In 2013 Li et al. (2013) reported that plasma levels of 9-HODE and 12-HETE were significantly higher in LDLR-null mice exposed to UFP compared with mice exposed to FA. Findings for plasma 13-HODE and 5-HETE were inconclusive, but mean concentrations were higher in mice exposed to UFP versus FA (*p* = 0.16 and 0.19 respectively). In a second study of UFP exposed LDLR-null mice by Li et al. (2015), significantly higher levels of 5-HETE, 12-HETE, 15-HETE, 9-HODE, 13-HODE, and PGD_2_ were reported in intestine and liver tissue of UFP-exposed mice compared with FA-exposed mice. In plasma, only 12-HETE, 15-HETE, and PGD_2_ levels were significantly higher when comparing UFP-exposed with FA-exposed mice. Reported higher plasma levels of 5-HETE, 9-HODE, and 13-HODE in UFP-exposed mice compared with FA-exposed mice were inconclusive (Li et al. 2015). These findings in animal studies are in line with our reported associations of *in utero* PM_2.5_ exposure with oxylipin profiles of the 5-LOX and 12/15-LOX pathway. Exposure during the second trimester was associated with cord blood plasma levels of 5-HETE, 12-HETE, 15-HETE, 9-HODE, and 13-HODE. However, the COX-derived PGD_2_ metabolite was not significantly associated with PM_2.5_ exposure during any time period in our study population (see Figure S2 and Table S6). Findings from the experimental studies, which used mouse models of atherosclerosis and high diesel exhaust and UFP exposures [e.g., 360 ± 25 μg/m^3^ of UFP in Li et al. (2015) compared with mean PM_2.5_ concentrations of 15.7 ± 2.6 μg/m^3^ in our study population], cannot be directly compared with the findings from our observational birth cohort study. Nonetheless, the experimental findings are generally consistent and support our results. Our findings, supported by these animal-based studies, suggests an important role of PM exposure in the alteration of the 5-LOX and 12/15-LOX pathways and derived oxylipins.

Our findings indicate possible early immunological alterations due to early-life exposure to particulate air pollution. Indeed, B-lymphocytes are able to produce the major 5-LOX metabolites 5-HETE and LTB_4_ in the presence of exogenous arachidonic acid and oxidative stress (Jakobsson et al. 1995). Grant et al. (2011) showed that the more biological active 5-oxoETE was produced in human B lymphocyte cell lines from its precursor 5-HETE after subjection to oxidative stress by H_2_O_2_. These findings suggest the ability of PM to induce oxidative stress by producing reactive oxygen species (ROS), which might stimulate the production of 5-HETE and 5-oxoETE in different types of leukocytes, reflecting a higher inflammatory state. However, the exact sources of the oxylipin metabolites measured in cord blood for our study are unknown.

Metabolites of the 12/15-LOX pathway have been indicated in pro- and anti-inflammatory responses (Uderhardt and Krönke 2012). Anti-inflammatory effects of 15-HETE and 13-HODE are related to their ability to modulate the activity of the peroxisome proliferator-activated receptor γ (PPARγ), implicated in adipocyte differentiation regulation, fatty acid storage, glucose metabolism, and anti-inflammatory processes (Martin 2010). In contrast to the involvement in anti-inflammatory properties, 15-HETE has been described in pro-inflammatory responses involved in air-way inflammatory diseases and asthma (Conrad 1999; Larsson et al. 2014; Lundström et al. 2011b; Sachs-Olsen et al. 2010). Our findings suggest that the second trimester of pregnancy is a very susceptible period for exposure in altering oxylipins. A potential explanation for this observation could be the fact that during pregnancy the syncytiothrophoblast layer, which forms the barrier between maternal and fetal blood, gets thinner with gestational age, and until week 10 of pregnancy the size of fetal capillaries increases leading to enhanced maternal fetal exchange of nutrients and particles (Proietti et al. 2013; Wick et al. 2010).

Strong points of our study are the high-resolution exposure estimates and integration of daily exposures to estimate pregnancy-specific exposure windows. The epidemiologic angle from the early-life perspective enhances the relevance of our findings for public health beyond that of studies in patients or older population segments that might be confounded by multiple comorbidities and polymedication. However, our present study must also be interpreted within the context of its limitations. Metabolomics is a top-down systems biology approach to understand the gene–environment–health paradigm. Cord blood is an important matrix to study metabolomic changes in the context of perinatal programming. However, we are able to study the associations only on changes in profiles at birth and not during pregnancy, which might be a limitation. Our sample size was relatively small, which might reduce the generalizability. Next, we analyzed oxylipin metabolites only in cord blood and not in maternal plasma during pregnancy, having only one snapshot at birth with no insight into the maternal link to the fetal oxylipin profiles. Keeping in mind the redundancy and bidirectionality in many metabolic pathways, studies such as ours cannot determine whether a metabolite is increased because of increased production, decreased degradation and/or uptake, or both. We also need to address the importance of maternal nutrition on the oxylipin state of the newborn, which may be an important factor influencing oxylipin levels that could not be taken into account in the present study. Essential fatty acids such as the ω-3 FA α-linolenic acid and ω-6 FA linoleic acid, which are precursors for oxylipin synthesis, have to be provided to the fetus by maternal nutrition (Herrera 2002). Because there exists a linear correlation between maternal intake of dietary fatty acids and maternal plasma and cord blood plasma concentrations of fatty acids, these might influence newborns’ oxylipin levels (Donahue et al. 2011; Herrera 2002). However, we do not expect exposure to air pollution to be associated with omega-fatty acid consumption in our study population, so it is unlikely that these nutritional aspects have confounded our reported associations. However, we cannot completely rule out unmeasured factors including nutrition that correlate with air pollution or its variation over seasons. Although we have used a standardized fine-scale exposure model for the estimation of residential fine particulate matter, maternal daily activity patterns could not be taken into account which contribute to maternal exposure levels. Furthermore, PM_2.5_ might present an epiphenomena or a proxy for exposure to other air pollutants. However, the observation that only PM_2.5_ was statistically significant in two-pollutant models (including both nitrogen dioxide and PM_2.5_) highlights the independent role of particles. However, we did not address to what extent specific constituents of the particulate matter were responsible for the observed associations. Finally, epidemiologic studies demonstrate association, and the interpretation of the association with the metabolic marker rests on review and interpretation of the literature, which involves some degree of speculation. Nevertheless, our observational findings in mother–newborn pairs add to existing knowledge in the field of effects of air pollution exposure on oxylipin levels.

## Conclusions

To our knowledge, our study is the first to report positive associations of metabolites from the 5-LOX and 12/15-LOX pathways in newborns with *in utero* exposure to PM_2.5_. These findings gain new insights in the pregnancy exposome and in the role of lipid mediators. Because metabolic profiles are more closely related to the phenotype compared with transcriptomic and proteomic profiles (Patti et al. 2012), these findings of PM-associated alterations of oxylipin profiles might have implications in later-life development of air pollution associated diseases. However, the consequences of alterations in early-life oxylipin profiles for later life diseases must be further elucidated in further prospective evaluations.

## Supplemental Material

(372 KB) PDF
