# Supplementary material for: Neonatal Cord Blood Oxylipins and Exposure to Particulate Matter in the Early-Life Environment: An ENVIRONAGE Birth Cohort Study
Source: Environ Health Perspect. 2016 Nov 4;125(4):691–8. doi: 10.1289/EHP291 (PMC5381981; doi:10.1289/EHP291)
Supplement: (372 KB) PDF [file EHP291.s001.acco.pdf]

Environ Health Perspect
DOI: 10.1289/EHP291

Note to readers with disabilities: *EHP* strives to ensure that all journal content is accessible to all readers. However, some figures and Supplemental Material published in *EHP* articles may not conform to 508 standards due to the complexity of the information being presented. If you need assistance accessing journal content, please contact ehponline@niehs.nih.gov. Our staff will work with you to assess and meet your accessibility needs within 3 working days.

Supplemental Material

Neonatal Cord Blood Oxylipins and Exposure to Particulate Matter in the Early-Life Environment: An ENVIR*ON*AGE Birth Cohort Study

Dries S. Martens, Sandra Gouveia, Narjes Madhloum, Bram G. Janssen, Michelle Plusquin, Charlotte Vanpoucke, Wouter Lefebvre, Bertil Forsberg, Malin Nording, and Tim S. Nawrot

Table of Contents

Chemical details for UPLC-MS/MS.

Table S1. Comparison characteristics of the ENVIR*ON*AGE data with data of the birth register of Flanders (Northern part of Belgium).

Table S2. Internal standard (IS) assignment to each native standard used for quantification.

Table S3. Calibration standard curves concentrations (pg/mL).

Table S4. Metabolite classification and description.

Table S5. Characteristics of the first derived principal component.

Table S6. Associations between oxylipin metabolites and different exposure windows of $PM_{2.5}$.

Figure S1. Heat map of correlations between the different quantified oxylipins from the four main different oxylipin biosynthetic pathways

Figure S2. Schematic representation of associations between individual oxylipin metabolites and different exposure windows of $PM_{2.5}$.

References

Chemical details for UPLC-MS/MS

The following native, internal, and recovery standards were purchased from Cayman Chemicals (Ann Arbor, MI, USA): $PGF_{2\alpha}$, PGE_2, TXB_2, PGD_2, 5(6)-EET, 8(9)-EET, 11(12)-EET, 14(15)-EET, 5,6-DHET, 8,9-DHET, 11,12-DHET, 14,15-DHET, 9(10)-EpOME, 12(13)-EpOME, 9(10)-DiHOME, 12(13)-DiHOME, 5-HETE, 8-HETE, 9-HETE, 11-HETE, 12-HETE, 15-HETE, 20-HETE, 9-HODE, 13-HODE, 15(S)-HETrE, 12-HEPE, 17-HDoHE, 5-oxo-ETE, 12-oxo-ETE, 15-oxo-ETE, 13-oxo-ODE, LTB_4, Resolvin D2, Resolvin D1, 12-[[(cyclohexylamino) carbonyl]amino]-dodecanoic acid (CUDA), 12(13)-DiHOME-d_4, 9-HODE-d_4, PGE_2-d_4, and TXB_2-d_4. The oxylipins 9,10,13-TriHOME and 9,12,13-TriHOME were obtained from Larodan (Sweden, Malmö). Acetonitrile and methanol were from Merck (Darmstadt, Germany). Isopropanol was purchased from VWR PROLABO (Fontenay-sous-Bois, France). Ammonium acetate was purchased from Scharlau Chemie (Barcelona, Spain). Acetic acid was purchased from Aldrich Chemical Company, Inc. (Milwaukee, WI, USA). Butylhydroxytoluene (BHT) was from Cayman Chemical (Ann Arbor, MI, USA) and ethylenediaminetetraacetic acid (EDTA) from Fluka Analytical, Sigma-Aldrich (Buchs, Switzerland). Glycerol was from Fischer Scientific (Loughborough, UK).

Table S1. Comparison characteristics of the ENVIR*ON*AGE data with data of the birth register of Flanders (Northern part of Belgium). This register comprise all birth from Flanders (*n*=648 711) from 1999-2009 (Cox et al. 2013). Values presented as percentage or as median (10-90th centile)

**Characteristic**	**ENVIR*ON*AGE (*n*=197)**	**Flanders (*n*=606 877)**
**Maternal age, years**	29 (23-35)	29.5 (23.5-35.8)
**Education**		
Low	13.7%	13.1%
Middle	38.6%	40.8%
High	47.7%	46.2%
**Parity**		
1	56.4%	46.9%
2	33.5%	34.7%
≥3	10.1%	18.4%
**Gender**		
Boys	54.8%	51.4%
**Ethnicity**		
European	85.3%	87.7%
**Birth weight, g**	3450 (2855-4010)	3360 (2740-3965)

Table S2. Internal standard (IS) assignment to each native standard used for quantification

Internal standard	Related native standard
TXB_2-d_4	TXB_2, 9,12,13-TriHOME, 9,10,13-TriHOME
12(13)-DIHOME-d_4	12(13)-DiHOME, 9(10)-DiHOME, 14,15-DHET, 11,12-DHET, 8,9-DHET, 5,6-DHET, 12(S)-HEPE, 20-HETE
9(S)-HODE-d_4	13-HODE, 9(S)-HODE, 15-HETE, 13-oxo-ODE, 11-HETE, 15-oxo-ETE, 9-oxo-ODE, 12-HETE, 8-HETE, 15(S)-HETrE, 12-oxo-ETE, 9-HETE, 5-HETE, 14(15)-EET, 11(12)-EET, 8(9)-EET, 5(6)-EET, 5-oxo-ETE, 9(10)-EpOME, 12(13)-EpOME
PGE_2-d_4	$PGF_{2\alpha}$, PGE_2, PGD_2, LTB_4

Table S3. Calibration standard curves concentrations (pg/mL)

Standards	Standard concentration (pg/mL)
**S1**	16450
**S2**	8220
**S3**	4110
**S4**	2060
**S5**	1030
**S6**	514
**S7**	257
**S8**	129
**S9**	64
**S10**	37

Table S4. Metabolite classification and description. Classification according to biosynthetic pathway and full name description with indication of fatty acid precursor (AA=arachidonic acid, LA=linoleic acid and EPA= eicosapentaenoic acid, DGLA= dihomo-γ-linolenic acid). Mean cord blood plasma oxylipin concentrations (in nM) and interquartile range (IQR) from 197 individuals and amount of individuals where the metabolite could not be detected. Limit of detection (LOD) provided for each metabolite in nM

Pathway	Metabolite	Full name	Precursor	mean (IQR)	# not detected	LOD (nM)*
COX	PGD_2	Prostaglandin D2	AA	0.2 (0.2)	2	0.015
	PGE_2	Prostaglandin E2	AA	1.2 (1.4)		0.0032
	$PGF_{2\alpha}$	Prostaglandin F2α	AA	1.6 (0.9)		0.0031
	TXB_2	Thromboxane B2	AA	3.2 (4.4)	1	0.0027
CYP	5,6-DHET	5,6-dihydroxy-eicosatrienoic acid	AA	0.4 (0.3)		0.0162
	8,9-DHET	8,9-dihydroxy-eicosatrienoic acid	AA	1.7 (1.2)		0.0162
	11,12-DHET	11,12-dihydroxy-eicosatrienoic acid	AA	1.4 (1.2)		0.0162
	14,15-DHET	14,15-dihydroxy-eicosatrienoic acid	AA	3.1 (1.8)		0.00325
	5(6)-EET	5(6)-epoxy-eicosatrienoic acid	AA	3.4 (4.2)		0.0811
	8(9)-EET	8(9)-epoxy-eicosatrienoic acid	AA	0.2 (0.3)	2	0.0156
	11(12)-EET	11(12)-epoxy-eicosatrienoic acid	AA	0.2 (0.3)		0.00312
	14(15)-EET	14(15)-epoxy-eicosatrienoic acid	AA	0.1 (0.2)	12	0.00312
	9,10-DiHOME	9,10-dihydroxy-octadecenoic acid	LA	0.9 (0.7)		0.00035
	12,13-DiHOME	12,13-dihydroxy-octadecenoic acid	LA	3.7 (6.3)		0.0035
	9(10)-EpOME	9(10)epoxy-octadecenoic acid	LA	0.6 (0.8)		0.0877
	12(13)-EpOME	12(13)epoxy-octadecenoic acid	LA	0.8 (1.0)		0.0877
	20-HETE	20-hydroxy-eicosatetraenoic acid	AA	0.5 (0.5)	6	0.1819
5-LOX	5-HETE	5-hydroxy-eicosatetraenoic acid	AA	0.8 (0.9)		0.0156
	5-oxoETE	5-oxo-eicosatetraenoic acid	AA	0.1 (0.1)	9	0.0157
	9(S)-HODE	9-hydroxy-octadecadienoic acid	LA	2.3 (2.3)		0.00037
	LTB_4	Leukotriene B4	AA	0.04 (0.04)	141	0.016
	9,10,13-TriHOME	9,10,13-trihydroxy-octadecenoic acid	LA	2.9 (4.2)		0.003
	9,12,13-TriHOME	9,12,13-trihydroxy-octadecenoic acid	LA	2.0 (2.7)		0.00015
12/15-LOX	8-HETE	8-hydroxy-eicosatetraenoic acid	AA	0.4 (0.4)		0.0312
	9-HETE	9-hydroxy-eicosatetraenoic acid	AA	0.3 (0.4)	46	0.0156
	11-HETE	11-hydroxy-eicosatetraenoic acid	AA	0.4 (0.4)		0.00171
	12-HETE	12-hydroxy-eicosatetraenoic acid	AA	3.5 (6.0)		0.0156
	12(S)-HEPE	12-hydroxy-eicosapentaenoic acid	EPA	0.2 (0.3)	4	0.0035
	12-oxoETE	12-oxo-eicosatetraenoic acid	AA	0.5 (0.6)	87	0.00312
	15-HETE	15-hydroxy-eicosatetraenoic acid	AA	0.5 (0.4)		0.0034
	15(S)-HETrE	15-hydroxy-eicosatrienoic acid	DGLA	0.3 (0.3)	1	0.0016
	15-oxoETE	15-oxo-eicosatetraenoic acid	AA	0.07 (0.07)	2	0.0157
	13-HODE	13-hydroxy-octadecadienoic acid	LA	3.7 (3.1)		0.0185
	13-oxoODE	13-oxo-octadecadienoic acid	LA	0.3 (0.3)	7	0.1692

Classification based on Zivkovic *et al*. (Zivkovic et al. 2012).

* LOD based on Gouveia-Figueira *et al*. (Gouveia-Figueira et al. 2015)

Table S5. Characteristics of the first derived principal component. Classification of principal components for each pathway with indications of individual metabolite loadings

Pathway PC	Eigenvalues	Variance explained	Metabolite	Loadings
PC: COX	1.94	48%	PGD_2	0.76
			PGE_2	0.82
			$PGF_{2\alpha}$	0.36
			TXB_2	0.74
PC: CYP	5.42	42%	5,6-DHET	0.71
			8,9-DHET	0.83
			11,12-DHET	0.87
			14,15-DHET	0.81
			5(6)-EET	0.54
			8(9)-EET	0.56
			11(12)-EET	0.29
			14(15)-EET	0.39
			9,10-DiHOME	0.80
			12,13-DiHOME	0.69
			9(10)-EpOME	0.62
			12(13)-EpOME	0.18
			20-HETE	0.66
PC: 5-LOX	2.57	43%	5-HETE	0.62
			5-oxoETE	0.61
			9(S)-HODE	0.76
			LTB_4	0.05
			9,10,13-TriHOME	0.76
			9,12,13-TriHOME	0.81
PC: 12/15-LOX	6.03	55%	8-HETE	0.83
			9-HETE	0.31
			11-HETE	0.93
			12-HETE	0.83
			12(S)-HEPE	0.63
			12-oxoETE	0.55
			15-HETE	0.90
			15(S)-HETrE	0.88
			15-oxoETE	0.64
			13-HODE	0.81
			13-oxoODE	0.59

Environ Health Perspect
DOI: 10.1289/EHP291

Table S6. Associations of oxylipin metabolites and different exposure windows of $PM_{2.5}$. Beta-coefficients presented for each 5 $\mu g/m^3$ increase of $PM_{2.5}$

	Trimester 1		Trimester 2		Trimester 3		Entire Pregnancy	
	β (95% CI)	*p*-Value	β (95% CI)	*p*-Value	β (95% CI)	*p*-Value	β (95% CI)	*p*-Value
**COX**								
PGD_2	-0.04 (-0.12, 0.04)	0.29	0.04 (-0.02, 0.09)	0.23	-0.02 (-0.10, 0.06)	0.55	0.02 (-0.10, 0.14)	0.75
PGE_2	-0.03 (-0.1, 0.03)	0.32	-0.02 (-0.07, 0.03)	0.42	-0.05 (-0.12, 0.02)	0.17	-0.08 (-0.18, 0.03)	0.15
$PGF_{2\alpha}$	-0.01 (-0.05, 0.02)	0.48	-0.02 (-0.04, 0.01)	0.22	-0.02 (-0.05, 0.02)	0.40	-0.05 (-0.1, 0.01)	0.09
TXB_2	-0.07 (-0.18, 0.04)	0.23	0.04 (-0.04, 0.12)	0.35	-0.08 (-0.20, 0.03)	0.17	-0.01 (-0.19, 0.17)	0.92
**CYP**								
5,6-DHET	-0.04 (-0.09, 0.01)	0.15	0.04 (0.00, 0.08)	0.08	-0.03 (-0.09, 0.03)	0.28	0.02 (-0.07, 0.11)	0.69
8,9-DHET	0.00 (-0.03, 0.04)	0.88	0.02 (-0.01, 0.05)	0.14	0.01 (-0.03, 0.05)	0.57	0.04 (-0.01, 0.10)	0.13
11,12-DHET	-0.01 (-0.05, 0.03)	0.65	0.03 (0.00, 0.07)	0.04	0.01 (-0.04, 0.05)	0.77	0.06 (-0.01, 0.13)	0.11
14,15-DHET	0.00 (-0.03, 0.03)	0.94	0.02 (-0.01, 0.04)	0.22	0.01 (-0.02, 0.04)	0.59	0.03 (-0.02, 0.08)	0.23
5(6)-EET	-0.03 (-0.11, 0.04)	0.38	0.04 (-0.01, 0.10)	0.13	-0.05 (-0.13, 0.03)	0.22	0.04 (-0.08, 0.16)	0.56
8(9)-EET	-0.02 (-0.11, 0.07)	0.74	0.02 (-0.05, 0.08)	0.60	-0.04 (-0.13, 0.06)	0.45	0.00 (-0.14, 0.14)	0.99
11(12)-EET	-0.05 (-0.12, 0.02)	0.14	0.03 (-0.02, 0.08)	0.25	-0.03 (-0.10, 0.05)	0.44	0.00 (-0.11, 0.11)	0.99
14(15)-EET	-0.04 (-0.18, 0.09)	0.53	0.02 (-0.08, 0.13)	0.63	-0.08 (-0.22, 0.06)	0.25	-0.03 (-0.25, 0.18)	0.78
9,10-DiHOME	-0.02 (-0.07, 0.03)	0.53	0.03 (-0.01, 0.06)	0.18	-0.01 (-0.07, 0.04)	0.65	0.03 (-0.05, 0.11)	0.43
12,13-DiHOME	-0.01 (-0.07, 0.04)	0.61	0.02 (-0.02, 0.06)	0.39	0.00 (-0.06, 0.06)	0.98	0.03 (-0.06, 0.12)	0.53
9(10)-EpOME	0.00 (-0.07, 0.07)	0.98	0.04 (-0.02, 0.09)	0.20	-0.03 (-0.10, 0.05)	0.49	0.05 (-0.06, 0.17)	0.37
12(13)-EpOME	-0.01 (-0.09, 0.06)	0.75	0.02 (-0.04, 0.08)	0.54	-0.02 (-0.11, 0.06)	0.55	0.01 (-0.11, 0.13)	0.89
20-HETE	-0.05 (-0.11, 0.01)	0.08	0.07 (0.02, 0.11)	0.004	-0.03 (-0.10, 0.03)	0.27	0.06 (-0.04, 0.16)	0.22
**5-LOX**								
5-HETE	0.00 (-0.06, 0.06)	0.99	0.05 (0.01, 0.09)	0.03	0.01 (-0.05, 0.07)	0.79	0.09 (0.00, 0.18)	0.05
5-oxoETE	0.06 (-0.04, 0.16)	0.22	0.08 (0.01, 0.15)	0.03	0.06 (-0.04, 0.16)	0.24	0.22 (0.07, 0.38)	0.006
9(S)-HODE	-0.01 (-0.07, 0.04)	0.64	0.05 (0.00, 0.10)	0.06	-0.02 (-0.08, 0.04)	0.51	0.07 (-0.04, 0.18)	0.20
LTB_4	0.04 (-0.03, 0.11)	0.30	-0.01 (-0.06, 0.05)	0.77	0.00 (-0.08, 0.07)	0.97	0.01 (-0.10, 0.13)	0.82
9,10,13-TriHOME	0.01 (-0.06, 0.08)	0.75	0.04 (-0.01, 0.08)	0.16	0.05 (-0.02, 0.12)	0.17	0.10 (-0.01, 0.20)	0.08
9,12,13-TriHOME	0.01 (-0.05, 0.07)	0.79	0.04 (-0.01, 0.09)	0.10	0.04 (-0.03, 0.10)	0.23	0.10 (0.00, 0.20)	0.05
**12/15-LOX**								
8-HETE	-0.02 (-0.09, 0.05)	0.63	0.06 (0.01, 0.11)	0.02	-0.03 (-0.1, 0.04)	0.39	0.09 (-0.02, 0.2)	0.12
9-HETE	0.04 (-0.10, 0.18)	0.58	0.06 (-0.04, 0.17)	0.22	-0.06 (-0.21, 0.08)	0.38	0.11 (-0.12, 0.33)	0.35
11-HETE	-0.02 (-0.08, 0.05)	0.58	0.06 (0.01, 0.10)	0.02	-0.01 (-0.08, 0.05)	0.66	0.08 (-0.02, 0.19)	0.10
12-HETE	-0.07 (-0.19, 0.05)	0.23	0.11 (0.02, 0.20)	0.02	-0.07 (-0.19, 0.06)	0.28	0.12 (-0.07, 0.32)	0.20
12(S)-HEPE	-0.06 (-0.18, 0.05)	0.28	0.11 (0.02, 0.19)	0.02	-0.08 (-0.21, 0.04)	0.17	0.12 (-0.07, 0.30)	0.22
12-oxoETE	-0.04 (-0.25, 0.18)	0.73	0.18 (0.01, 0.34)	0.03	-0.04 (-0.26, 0.19)	0.76	0.28 (-0.06, 0.63)	0.11
15-HETE	-0.01 (-0.07, 0.06)	0.83	0.07 (0.02, 0.11)	0.006	-0.02 (-0.09, 0.04)	0.51	0.10 (0.00, 0.20)	0.04
15(S)-HETrE	-0.02 (-0.10, 0.06)	0.64	0.06 (0.00, 0.12)	0.04	-0.02 (-0.10, 0.07)	0.72	0.09 (-0.03, 0.22)	0.15
15-oxoETE	-0.02 (-0.10, 0.06)	0.59	0.04 (-0.02, 0.10)	0.16	-0.01 (-0.09, 0.07)	0.80	0.06 (-0.07, 0.19)	0.36
13-HODE	-0.02 (-0.09, 0.04)	0.48	0.05 (0.00, 0.09)	0.05	-0.03 (-0.09, 0.04)	0.44	0.06 (-0.04, 0.16)	0.26
13-oxoODE	0.01 (-0.07, 0.09)	0.79	0.03 (-0.03, 0.09)	0.27	-0.03 (-0.11, 0.06)	0.53	0.06 (-0.07, 0.18)	0.38

Environ Health Perspect
DOI: 10.1289/EHP291

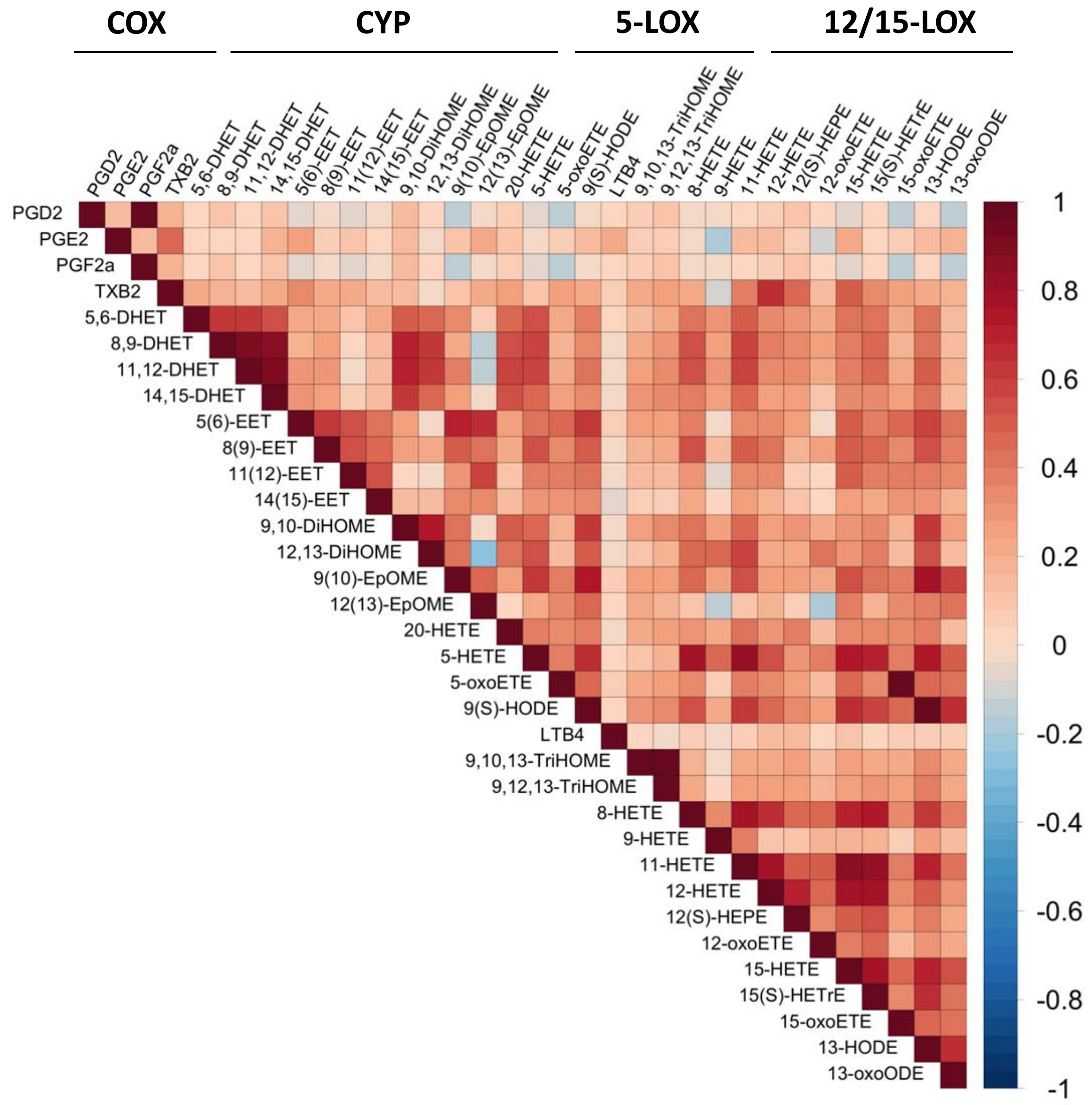

Figure S1. Heat map of correlations between the different quantified oxylipins from the four main different oxylipin biosynthetic pathways. Each colored square represents the Pearson correlation coefficient between different metabolites.

DOI: 10.1289/EHP291

	$PM_{2.5}$ Exposure window			
	Trim 1	Trim 2	Trim 3	Entire Pregnancy
COX				
PGD_2				
PGE_2				
$PGF_{2\alpha}$				-
TXB_2				
CYP		+		
5,6-DHET		+		
8,9-DHET				
11,12-DHET		+		
14,15-DHET				
5(6)-EET				
8(9)-EET				
11(12)-EET				
14(15)-EET				
9,10-DiHOME				
12,13-DiHOME				
9(10)-EpOME				
12(13)-EpOME				
20-HETE	-	+		
5-LOX		+		+
5-HETE		+		+
5-oxoETE		+		+
9(S)-HODE		+		
LTB4				
9,10,13-TriHOME				+
9,12,13-TriHOME		+		+
12/15LOX		+		+
8-HETE		+		
9-HETE				
11-HETE		+		+
12-HETE		+		
12(S)-HEPE		+		
12-oxoETE		+		
15-HETE		+		+
15(S)-HETrE		+		
15-oxoETE				
13-HODE		+		
13-oxoODE				

Figure S2. Schematic representation of association between individual oxylipin metabolites and different exposure windows of $PM_{2.5}$. Positive and negative associations are indicated by + and – signs respectively. Plain grey indicates p<0.05 and grey striped indicates 0.05<p<0.1

DOI: 10.1289/EHP291

References

Cox B, Martens E, Nemery B, Vangronsveld J, Nawrot TS. 2013. Impact of a stepwise introduction of smoke-free legislation on the rate of preterm births: Analysis of routinely collected birth data. BMJ 346:f441.

Gouveia-Figueira S, Nording ML, Gaida JE, Forsgren S, Alfredson H, Fowler CJ. 2015. Serum levels of oxylipins in achilles tendinopathy: An exploratory study. PLoS One 10:e0123114.
Zivkovic AM, Yang J, Georgi K, Hegedus C, Nording ML, O'Sullivan A, et al. 2012. Serum oxylipin profiles in iga nephropathy patients reflect kidney functional alterations. Metabolomics 8:1102-1113.
